# Quantitative assessments of finger individuation with an instrumented glove

**DOI:** 10.1186/s12984-023-01173-0

**Published:** 2023-04-20

**Authors:** Brian J. Conway, Léon Taquet, Timothy F. Boerger, Sarah C. Young, Kate B. Krucoff, Brian D. Schmit, Max O. Krucoff

**Affiliations:** 1 Medical College of Wisconsin, 8701 W Watertown Plank Rd, Milwaukee, WI 53226 USA; 2 Department of Neurosurgery, Medical College of Wisconsin, Milwaukee, WI USA; 3 Department of Plastic Surgery, Medical College of Wisconsin, Milwaukee, WI USA; 4 Department of Biomedical Engineering, Marquette University and Medical College of Wisconsin, Milwaukee, WI USA

**Keywords:** Finger individuation, Hand dexterity, Neuro-engineering, Motor systems, Cyberglove, Kinematics, Neurophysiology

## Abstract

**Background:**

In clinical and research settings, hand dexterity is often assessed as finger individuation, or the ability to move one finger at a time. Despite its clinical importance, there is currently no standardized, sufficiently sensitive, or fully objective platform for these evaluations.

**Methods:**

Here we developed two novel individuation scores and tested them against a previously developed score using a commercially available instrumented glove and data collected from 20 healthy adults. Participants performed individuation for each finger of each hand as well as whole hand open-close at two study visits separated by several weeks. Using the three individuation scores, intra-class correlation coefficients (ICC) and minimal detectable changes (MDC) were calculated. Individuation scores were further correlated with subjective assessments to assess validity.

**Results:**

We found that each score emphasized different aspects of individuation performance while generating scores on the same scale (0 [poor] to 1 [ideal]). These scores were repeatable, but the quality of the metrics varied by both equation and finger of interest. For example, index finger intra-class correlation coefficients (ICC’s) were 0.90 (< 0.0001), 0.77 (< 0.001), and 0.83 (p < 0.0001), while pinky finger ICC’s were 0.96 (p < 0.0001), 0.88 (p < 0.0001), and 0.81 (p < 0.001) for each score. Similarly, MDCs also varied by both finger and equation. In particular, thumb MDCs were 0.068, 0.14, and 0.045, while index MDCs were 0.041, 0.066, and 0.078. Furthermore, objective measurements correlated with subjective assessments of finger individuation quality for all three equations (ρ = − 0.45, p < 0.0001; ρ = − 0.53, p < 0.0001; ρ = − 0.40, p < 0.0001).

**Conclusions:**

Here we provide a set of normative values for three separate finger individuation scores in healthy adults with a commercially available instrumented glove. Each score emphasizes a different aspect of finger individuation performance and may be more uniquely applicable to certain clinical scenarios. We hope for this platform to be used within and across centers wishing to share objective data in the physiological study of hand dexterity. In sum, this work represents the first healthy participant data set for this platform and may inform future translational applications into motor physiology and rehabilitation labs, orthopedic hand and neurosurgery clinics, and even operating rooms.

## Introduction

Hands are complex components of the human motor system essential for a range of tasks, from simple grasping of objects to immensely intricate performances like art, music, and surgery. The ability to execute these movements hinges on hand dexterity [1–4], or the ability to use complex motor functions to manipulate objects on a small scale. Because numerous neurological resources are required, the process is quite susceptible to damage and dysfunction [5, 6]. Coordinated motor planning is thought to begin across a network of distributed cortical brain areas, eventually coalescing into activation of neurons in layer 5 of the hand and finger area of the primary motor cortex (M1). Action potentials from these neurons propagate down corticospinal tracts onto anterior horn and interneuronal cells of the spinal cord [7]. Anterior horn cells in turn become peripheral nerves traveling through the brachial plexus and down the arm, eventually innervating muscles of the hand and forearm to move a finger [7, 8]. This motor pathway is integrated with multimodal afferent sensory pathways in an even more complex, non-linear, hierarchical fashion [9, 10].

Injuries impacting hand function can entail complex processes occurring anywhere from the brain to spinal cord to peripheral nerves or intrinsic muscles. While clinicians are generally able to identify gross changes in motor function, injury, intervention, and recovery are physiologically multifaceted processes necessitating metrics sensitive enough to detect fine changes in dexterity which can impact quality of life [3, 4, 6, 11–18]. Therefore, there is a need for an objective, quantitative hand function assessment platform to better guide its study and intricate neurological interventions, such as brain and peripheral nerve surgery [7, 13, 14, 19–24].

Currently, hand dexterity is often assessed in clinical and research settings as kinematic finger individuation [25, 26], as these abilities have been shown to concomitantly diminish in patients with brain injury [2]. Unfortunately, such assessments are often subjective and not sufficiently sensitive to detect subtle problems. While other groups have provided foundational work developing objective kinematic individuation scores as a technique of quantifying hand dexterity in non-human primates [26] and individuals with stroke [1, 25, 27, 28], there is an opportunity to explore more nuanced aspects of movement relevant to different clinical circumstances required for more broad translational research applications. Additionally, there is limited published data on kinematic individuation in healthy adults [1, 25, 26]. Such data is essential for a complete understanding of the assessment method and future comparisons to patients with hand motor deficits. Therefore, here we present two novel individuation scores that emphasize different aspects of finger individuation by weighting various aspects of movement, like extreme ranges of motion versus midrange co-movement. We used these equations along with a previously published Eq. (1) to calculate individuation scores in 20 healthy volunteers with a commercially available data glove across repeated sessions. We include assessments of repeatability, minimal detectable change (MDC), and subjective score-performance evaluations using this platform. We hope that these results set the stage for the informed translational application of this objective data glove platform and scoring system into motor physiology and rehabilitations labs, orthopedic hand and neurosurgery clinics, and even intraoperative arenas during peripheral nerve and awake brain operations.

## Methods

### Ethical approval and participants

20 adult participants were recruited (16 female, 4 male, all right hand dominant) (Table 1) who met the following inclusion criteria: (1) age 18 + , (2) ability to understand a written informed consent document and the willingness to sign it, (3) normal or near normal hand motor strength (i.e., 5/5 on the manual motor scale), (4) normal or near normal speech, (5) free of other illness that in the judgement of the investigators may shorten life expectancy, (6) willing and able to participate in all aspects of the study. Participants were ineligible if they met any of the following exclusion criteria: (1) history or presence of malignancy within the last 3 years, except participants who have been successfully treated with no recurrence for > 1 year of basal cell or squamous cell carcinoma of the skin or in-situ cervical cancer, (2) decreased hand motor strength (i.e. 4/5), (3) atypical form of the hand interfering with their ability to perform the different hand movements, (4) any other significant pre-existing medical conditions that in the judgement of the investigators may increase the risks associated with study participation or would preclude successful participation in the study. Written informed consent was obtained from all participants. This study was conducted with approval from the Medical College of Wisconsin (MCW) Institutional Review Board (IRB) (PRO00040521) and the Froedtert Health Office of Clinical Research and Innovative Care Compliance (OCRICC).

**Table 1:** Healthy Adult Participant Characteristics

Participant Characteristics		
Sex	Female	16 (80%)
	Male	4 (20%)
Handedness	Right	20 (100%)
	Left	0 (0%)
Age	Mean ± SD	28.8 ± 7.5
	Median	27.5
	Minimum	23
	Maximum	55

### Kinematic individuation tasks

**Fig. 1:**
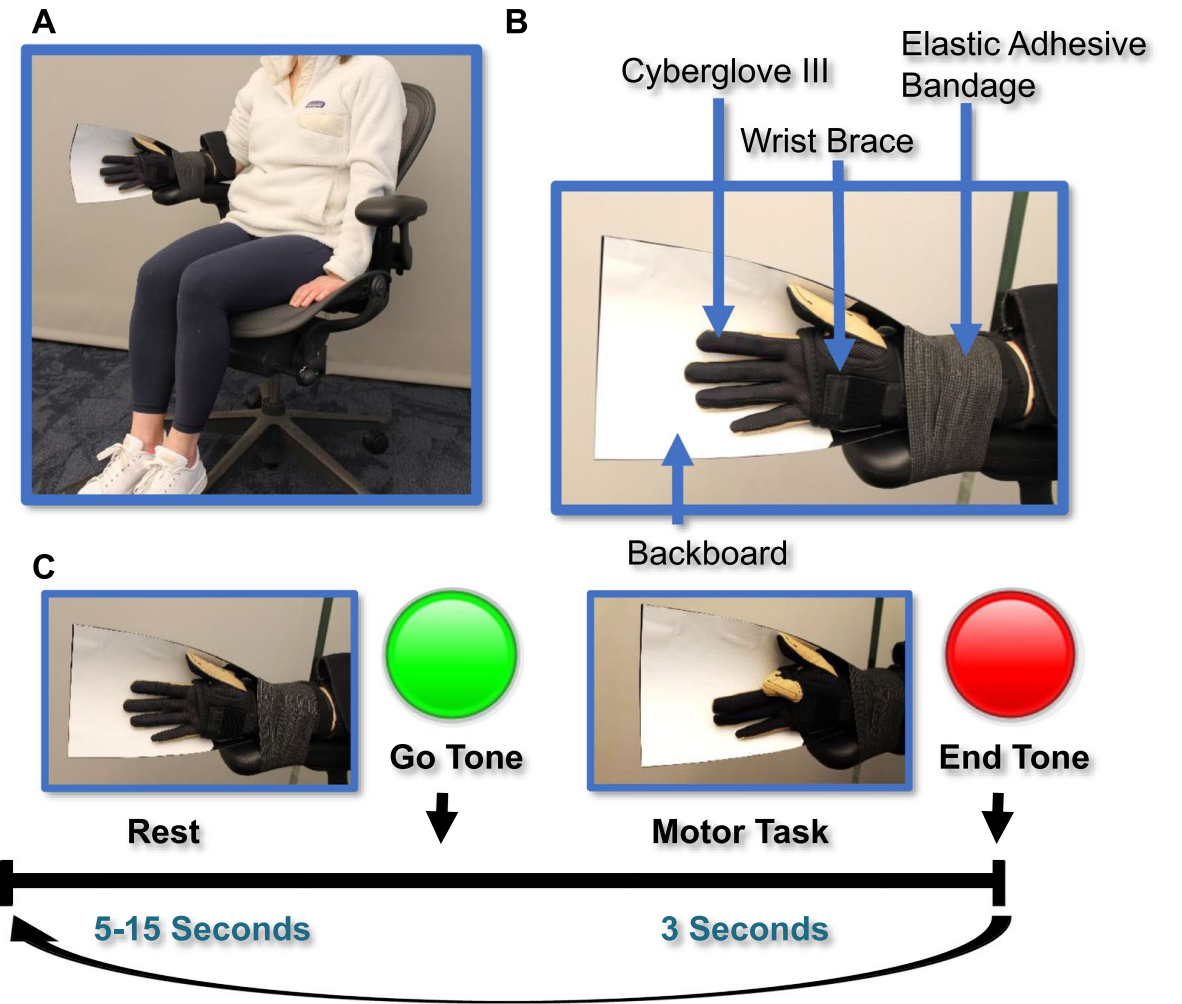
Experimental Protocol. **A**, **B** Participants were positioned as shown while recording data from each hand. **C** Photos of a participants with their hand at rest and while performing kinematic individuation and a timeline of events

Data were collected on each hand individually at two visits separated by at least three weeks. Participants wore the Cyberglove III (CyberGlove Systems, San Jose, CA) while sitting in an office chair (Fig. 1A). The Cyberglove III has 22 strain gauge resistors capable of translating a voltage to the joint angles of the hand at a sampling rate of 90 Hz. It has been used in many other neuroscience studies investigating hand movements [25, 29, 30]. All data analysis and calculation of individuation scores was performed in MATLAB (2021).

The forearm was immobilized by attaching it to the arm of the chair with self-adherent elastic wrap (Fig. 1A, B). Additionally, participants wore a simple wrist brace (in either the small/medium or medium/large size as applicable) during testing to maintain their hand in a resting neutral position and to control for involvement of forearm muscles as much as possible (Fig. 1A, B). To standardize participants’ resting position between movement trials, a backboard of heat-moldable plastic (11″ × 7″) shaped into a curved position to represent a generally neutral position was attached to the back of participants’ hands with self-adherent elastic wrap (Fig. 1A, B). The same backboard was used for all visits and participants.

At the two data collection visits, participants performed kinematic individuation where they were instructed to maximally flex an indicated finger without moving the other fingers. Participants performed 10 trials with each finger on each hand, and the order in which hands and fingers were tested was randomized prior to enrolling the participant. Additionally, participants performed 10 trials of closing their hand into a fist with their thumb on the outside and opening it again, and these data were also used to calculate individuation scores to further validate the equations. Participants viewed a screen with example pictures of the hand at rest and fully individuated positions. To indicate the start of a trial, participants heard a go-tone and a light changed from red to green. Participants performed the movement within a three second time frame paced by a visual cue. When the trial ended, the light changed back to red (Fig. 1C).

### Finger individuation scores

To assess participants’ individuation abilities, we used one previously developed and two original kinematic individuation equations. All equations were designed to generate a score between 0 and 1 with 1 representing the theoretically “ideal” individuation (i.e., maximally moving only the joints of the indicated finger and none of others) and 0 representing the theoretically “poorest” individuation (i.e., moving all fingers together). Trials where participants incorrectly performed movements and/or misunderstood directions were excluded from analysis, which was approximately one to three trials per study visit per participant. Scores calculated with the previously developed, or “Thielbar”, equation (Eq. 2) and the two original normalized (Eq. 3) and threshold equations (Eq. 4), which place an emphasis on end- and mid-range of motion, respectively, were investigated.

**Fig. 2:**
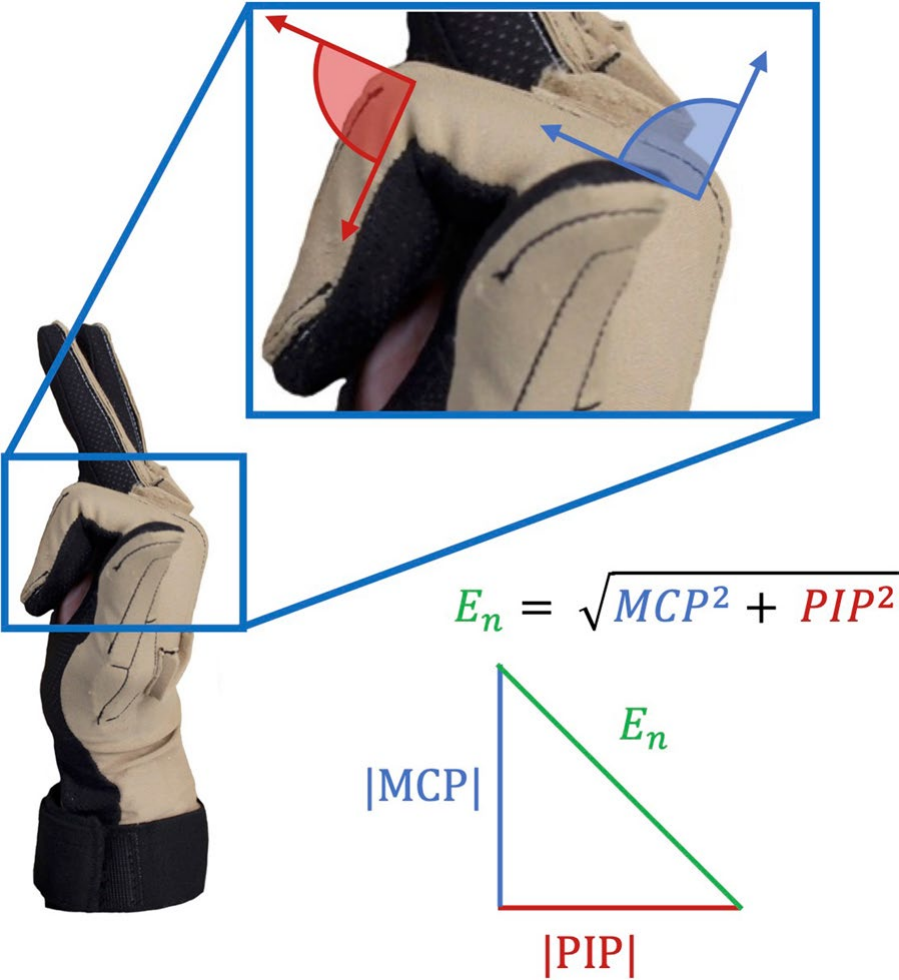
Euclidean Norm of Finger Movement. The metacarpophalangeal (MCP) (blue) and proximal interphalangeal (PIP) (red) joint angles were combined to calculate Euclidean norms (green) for each finger

In all three equations, individual finger displacement was calculated as the Euclidean norm of its metacarpophalangeal (MCP) and proximal interphalangeal (PIP) joint angles (Fig. 2, Eq. 1) [25]. Distal interphalangeal joint (DIP) movement was not incorporated into this calculation as its measurement was inconsistent and not as clinically important [25]. Utilization of Euclidean norms rather than MCP or PIP joint angle alone offers the advantage of accounting for different strategies of individuation as some participants may engage one joint more than the other in executing this movement to the best of their ability [25].

*Euclidean norm of finger movement*

1 $${E}_{n}= \sqrt{{MCP}^{2}+{PIP}^{2}}$$

*E*_*n*_ Euclidean norm, *MCP* metacarpophalangeal joint angle, *PIP* proximal interphalangeal joint angle.

To assess repeatability, intra-class correlation coefficients (ICC) were calculated by correlating participants’ mean individuation scores across visits for each finger [31]. Bland–Altman plots were then constructed to visualize this relationship [32]. The minimal detectable change (MDC) was also calculated.

*Thielbar Individuation Score (1)*

2 $$Score=1- \frac{\left[\frac{{E}_{ni}}{Max({E}_{ni})}+ \sum \frac{{E}_{no}}{Max({E}_{no})}\right]-1}{4}$$

*E*_*ni*_ Euclidean norm of the indicated finger, *E*_*no*_ Euclidean norm of a non-indicated finger.

**Fig. 3:**
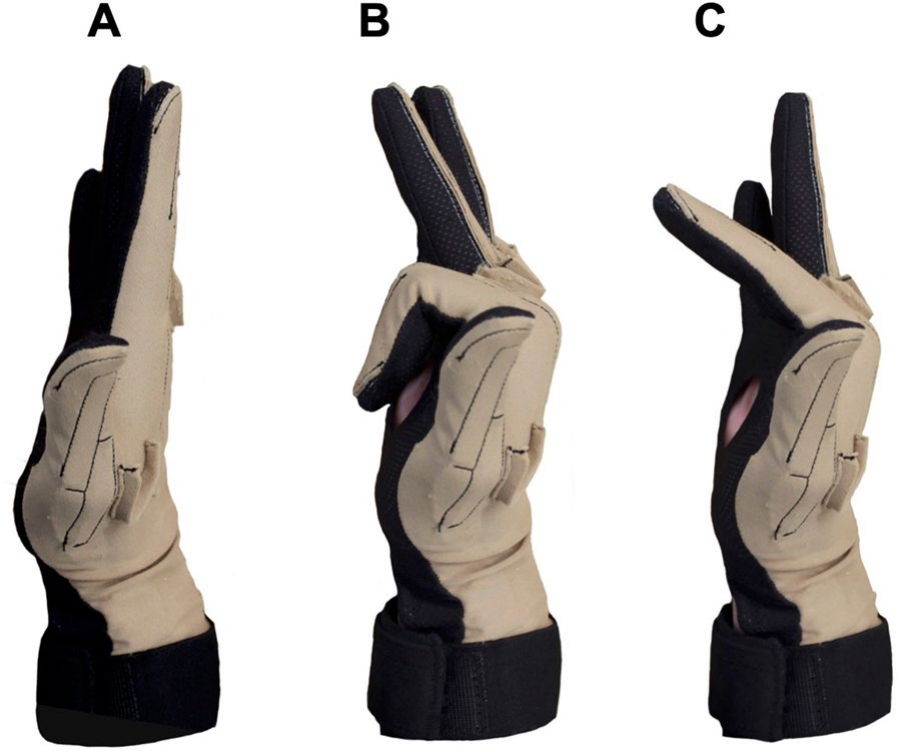
Relevant Hand Positions. Key Euclidean norms needed for the three individuation scores include the hand at rest (**A**), with the indicated finger fully flexed (**B**), and with the indicated finger reaching the 50% threshold (**C**)

The “Thielbar” individuation equation (Eq. 2) was developed by Thielbar and colleagues in their study on hand dexterity in stroke patients, (1) which built upon foundational work by Schieber and colleagues [1, 26, 27]. Utilizing this equation requires identification of the greatest Euclidean norm achieved by the indicated digit (E_ni_) as well as identification of the Euclidean norms of the four other non-indicated digits when the indicated digit is maximally flexed (E_no_) (Fig. 3B). The identified Euclidean norms of the indicated digit are then normalized to the overall maximum Euclidean norm achieved by each digit across all 10 trials (Max(E_ni_), and those of the non-indicated digit are normalized to the maximum Euclidean norms achieved when they were each the indicated digit Max(E_no_). This equation follows a template established by Schieber (1991) where he indicated that 1 must be subtracted from the numerator of the second term in Eq. 1 “to eliminate the contribution of the [displacement] of the instructed digit against itself.” [25, 26]. The numerator of the second term is then divided by 4 to account for the average contribution of the 4 non-indicated digits. The entire second term is then subtracted from 1, since 1 is the theoretically “perfect” individuation score [26].

*Normalized individuation score*

3 $$Score= \frac{\left|{E}_{ni}-{E}_{nib}\right|}{Max (\left|{E}_{ni}-{E}_{nib}\right|)}-\frac{\sum \frac{\left|{E}_{no}-{E}_{nob}\right|}{Max\left(\left|{E}_{no}-{E}_{nob}\right|\right)}}{4}$$

*E*_*nib*_ Euclidean norm of the indicated finger’s baseline position prior to initiation of movement, *E*_*nob*_ Euclidean norm of a non-indicated finger’s baseline position.

We developed the “normalized” individuation equation to account for variations in achievable ranges of motion across digits and individuals, thereby normalizing the achieved range of motion in each trial to the individual’s own maximum achievable range. This is important as end-of-range movements is often when non-indicated digits begin to move, so we therefore reward participants for a fuller-range attempt without moving other fingers. In other words, this equation translates a higher percent maximum range of motion per trial to a higher individuation score. This is especially relevant to pathological conditions, such as osteoarthtitis, where someone might have a baseline offset and limited range of motion. The normalized individuation equation will likely limit the impact of conditions such as these on individuation scores, since they are not related to the ability to execute the movement itself.

Similar to the Thielbar individuation equation, the normalized individuation equation requires identification of the maximum Euclidean norm of the indicated digit in a given trial (E_ni_) (Fig. 3B). However, a difference in this equation is that the baseline position of the indicated digit (E_nib_) (Fig. 3A) is subtracted from E_ni_ and then normalized to the overall maximum Euclidean norm digit displacement achieved across all 10 trials at a given visit (Max|E_ni_ – E_nib_|) for an indicated finger (i.e., maximum achievable range for that digit). This first term in the normalized individuation equation is typically nearly equal to 1, as healthy participants can easily approach their maximum range of motion. The second term is calculated as the sum of the Euclidean norm displacement of the 4 other non-indicated fingers (|E_no_-E_nob_|) normalized to the maximum Euclidean norm displacement achieved by each finger when it was the indicated digit (Max(|E_no_ – E_nob_|). The numerator of the second term is divided by 4, thus representing the average displacement of the non-indicated digits during a trial. The entire second term is subtracted from the first term. Therefore, if the non-indicated digits are moved extraneously, the second term will be greater, and the overall individuation score from the normalized individuation equation will be lower. Conversely, if the non-indicated digits are minimally moved, the score will remain close to 1. Moreover, achieving greater range of motion in a given trial that is close to a participants’ maximum ability results in an overall higher score – thus, the score from this equation will improve with increased range of motion.

*Threshold individuation score*

4 $$Score= \frac{\left|{E}_{nit}-{E}_{nib}\right|}{\left|{E}_{nit}-{E}_{nib}\right|}-\frac{\sum \frac{\left|{E}_{not}-{E}_{nob}\right|}{Max\left(\left|{E}_{thresh}-{E}_{nob}\right|\right)}}{4}$$

*E*_*nit*_ = Euclidean norm threshold of the indicated finger: 50% of the sample’s mean maximum Euclidean norm achieved by the indicated finger, *E*_*not*_ = Euclidean norm threshold of a non-indicated finger: position of the non-indicated digit when the indicated digit reaches E_nit_, *E*_*thresh*_ = Euclidean norm threshold for each of the non-indicated fingers.

Although accounting for maximal range of motion in calculating individuation scores offers unique insight into an individual’s dexterity, some may be severely impaired or have low baseline dexterity resulting in only having the capacity to perform basic components of the movement. Therefore, the “threshold” individuation equation (Eq. 4) was developed to capture an individual’s ability to reach 50% of the group’s mean range of motion with the indicated digit (E_nit_) (Fig. 3C). The E_nit_ of each digit was determined by identifying the mean of the study sample’s maximum Euclidean norm and calculating 50% of that value. We then verified that all participants’ mean maximum Euclidean norm exceeded these thresholds. In a given trial, the E_nit_ was determined by identifying the Euclidean norm closest to the pre-determined 50% threshold. The baseline Euclidean norm (E_nib_) (Fig. 3A) was subtracted from the indicated digit and then normalized to itself making the first term always equal to one across all trials. The second term of the threshold individuation equation resembles that of the normalized individuation equation, but it includes the Euclidean displacement achieved by the non-indicated digits when the indicated digit reaches the pre-specified threshold (|E_not_ – E_nob_|). This displacement of the non-indicated fingers is then normalized to their respective Euclidean norm thresholds (E_thresh_) minus the baseline position (E_nob_). Similar to the normalized individuation equation, the numerator of the second term is divided by 4 to quantify average displacement of the non-indicated fingers. Thus, the threshold individuation equation offers a quantified assessment of how well a participant can perform 50% of the kinematic individuation task.

### Subjective assessment and individuation score validation

To validate the individuation scores developed here, and because kinematic individuation has traditionally been subjectively assessed [33–35], a subset of individuation trials was also subjectively scored by two independent reviewers, the lead author (BC) and middle author (TB), a licensed athletic trainer with a PhD in Exercise and Rehabilitative Sciences. These individuals watched videos of the participants performing 10 trials of individuation of the right index and pinky fingers at participants’ first study visit. The scoring method used was developed by our multidisciplinary author group to stratify individuation performance using objective criteria that correlated with subjective assessments of what constitutes “good” individuation. To this end, each trial was given a rating of 1, 2, or 3 using the following scale: 1-excellent, less than 45 degrees of movement of any of the non-indicated fingers; 2-moderate, greater than 45 degrees of one non-indicated finger; 3-poor, greater than 45 degrees of more than one non-indicated finger. Movements of the index and pinky fingers were chosen to be reviewed as these were generally associated with participants’ best and worst individuation scores, respectively. Once both reviewers viewed videos from all participants, any conflicts in subjective rating were resolved. Both index and pinky finger trials from six participants and the index finger trials from two participants were excluded from the subjective ratings validation due to compromised video recordings. The subjective ratings on a scale from 1 to 3 were correlated to the corresponding individuation score calculated from each of the three individuation equations, and Spearman’s rho was calculated to assess the relationship between a subjective perception of the quality of movement and a quantified evaluation via individuation score.

Additionally, individuation scores were calculated from the data collected while participants performed the 10 trials of closing their hand into a fist and opening again. Since the aim of this movement is to move all fingers, whichever finger had the greatest range of motion was selected as the ‘indicated’ finger and the others were the non-indicated fingers for the Thielbar and normalized individuation scores (Eqs. 2, 3). For the threshold scores (Eq. 3), whichever finger reached the 50% threshold first was selected as the indicated finger with the others as the non-indicated fingers. In theory, this should represent the worst possible individuation performance.

## Results

### Task visualization

**Fig. 4:**
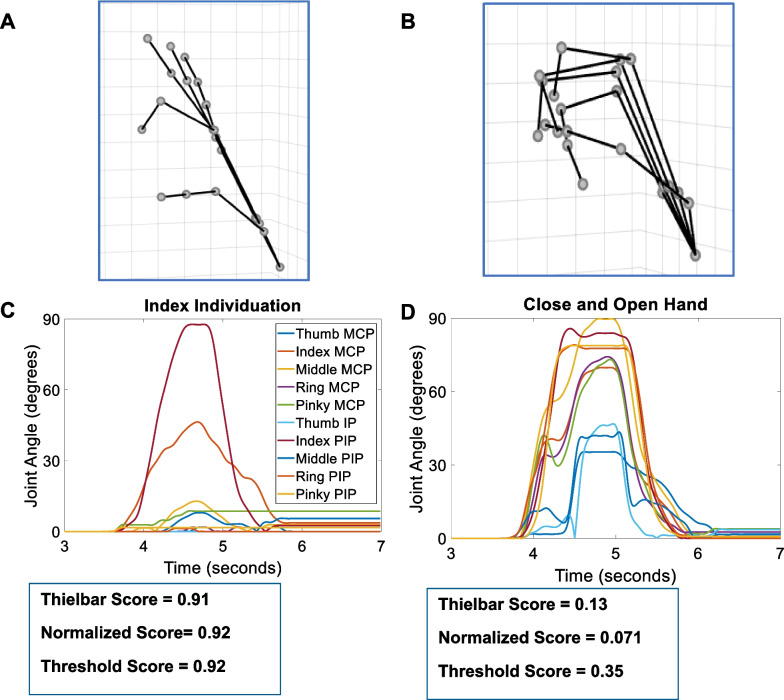
Example data collected with the Cyberglove III in a single participant. Three-dimensional reconstructions of the Cyberglove data while performing index individuation (**A**) and closing the hand into a fist (**B**). Joint angle traces while performing index individuation (**C**) and closing the hand into a fist (**D**) and the corresponding individuation scores

Three-dimensional reconstructions were developed from the Cyberglove III joint angle data to visualize individual trials and confirm the data accurately depicted the assigned kinematic task (Fig. 4A, B). Additionally, plots showed the joint angle traces of kinematic tasks for both individuation (Fig. 4C) and closing the hand into a fist (Fig. 4D) to observe the relationship between finger movements and corresponding individuation scores. Figure 4C shows an example trial of index finger individuation with clear movement of the index MCP and PIP joints and minimal movements of others with Thielbar, normalized, and threshold individuation scores of 0.91, 0.92, and 0.92, respectively. A similar graphical representation of closing the hand into a fist is presented in Fig. 4D with all joint angles increasing as the hand closes and returning to baseline as the hand opens. The Thielbar, normalized, and threshold individuation equations scored these movements as 0.13, 0.071, and 0.35, respectively (Fig. 4D).

### Distribution of individuation scores

**Fig. 5:**
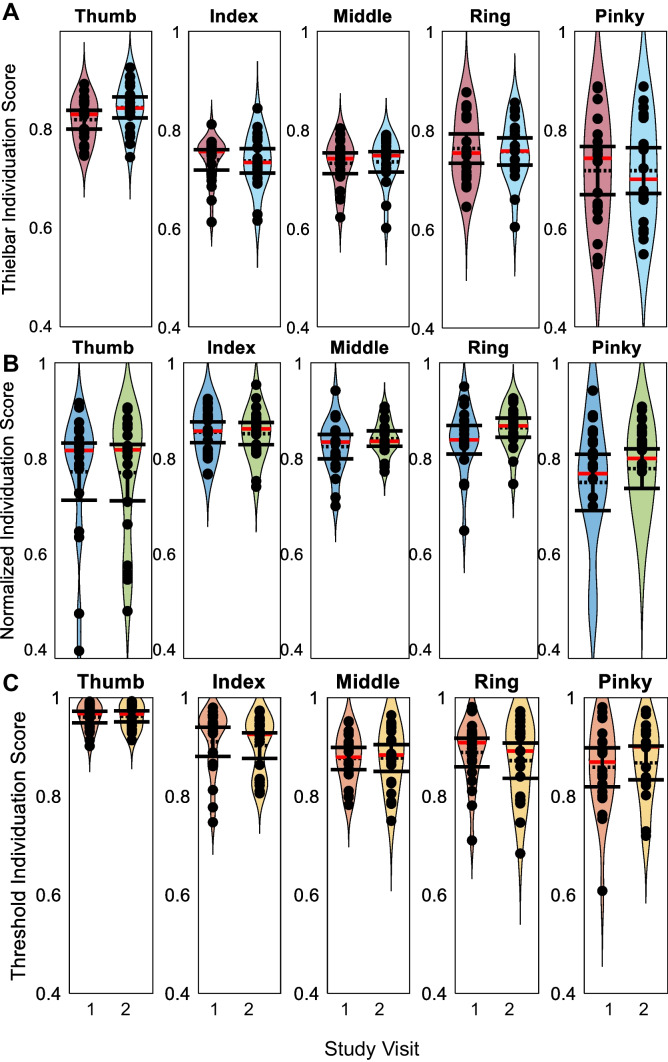
Distribution of individuation scores. Thielbar (**A**), normalized (**B**), and threshold (**C**) individuation scores of the thumb, index, middle, ring, and pinky fingers fall into unique ranges and have non-normal distributions. Overlapping 95% confidence intervals (shown as error bars) demonstrate the repeatability of kinematic individuation. Points represent participants’ mean individuation scores. Sample means are shown as a black dashed line. Sample medians are represented as red lines. Data from first visits are shown on the left in the graphs for each finger in the colors maroon, blue, and orange. Data from second visits are on the right in the colors cyan, green, and yellow

For all three individuation equations, participants’ mean scores fell into partially overlapping ranges and followed non-normal distributions for both study visits. Participants’ mean Thielbar individuation scores across all fingers and both visits ranged from 0.60 to 0.92 (Fig. 5A). Mean normalized individuation scores across all fingers and both visits ranged from 0.40 to 0.95 (Fig. 5B). Mean threshold individuation scores across all fingers and visits ranged from 0.68 to 0.99 (Fig. 5C). Overlapping 95% confidence intervals between the two visits for all fingers and equations are noted (Fig. 5).

### Repeatability of individuation scores

**Fig. 6:**
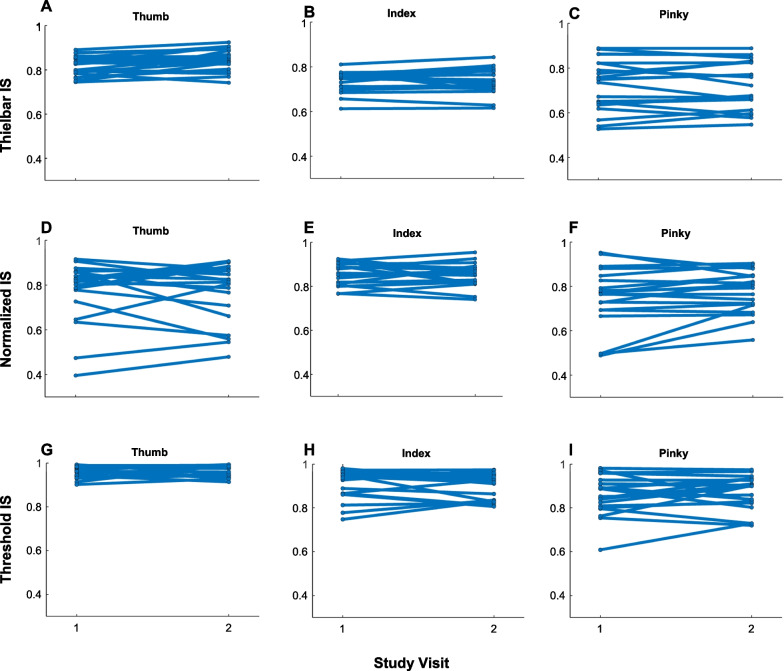
Participants’ Individuation scores between two visits. There was minimal change in participants’ mean Thielbar (**A**–**C**), Normalized (**D**–**F**), and Threshold (**G**–**I**) individuation scores (IS) between the two visits for all fingers. The thumb, index, and pinky fingers are shown here as the thumb and index were typically participants’ highest scores while the pinky was the lowest

**Fig. 7:**
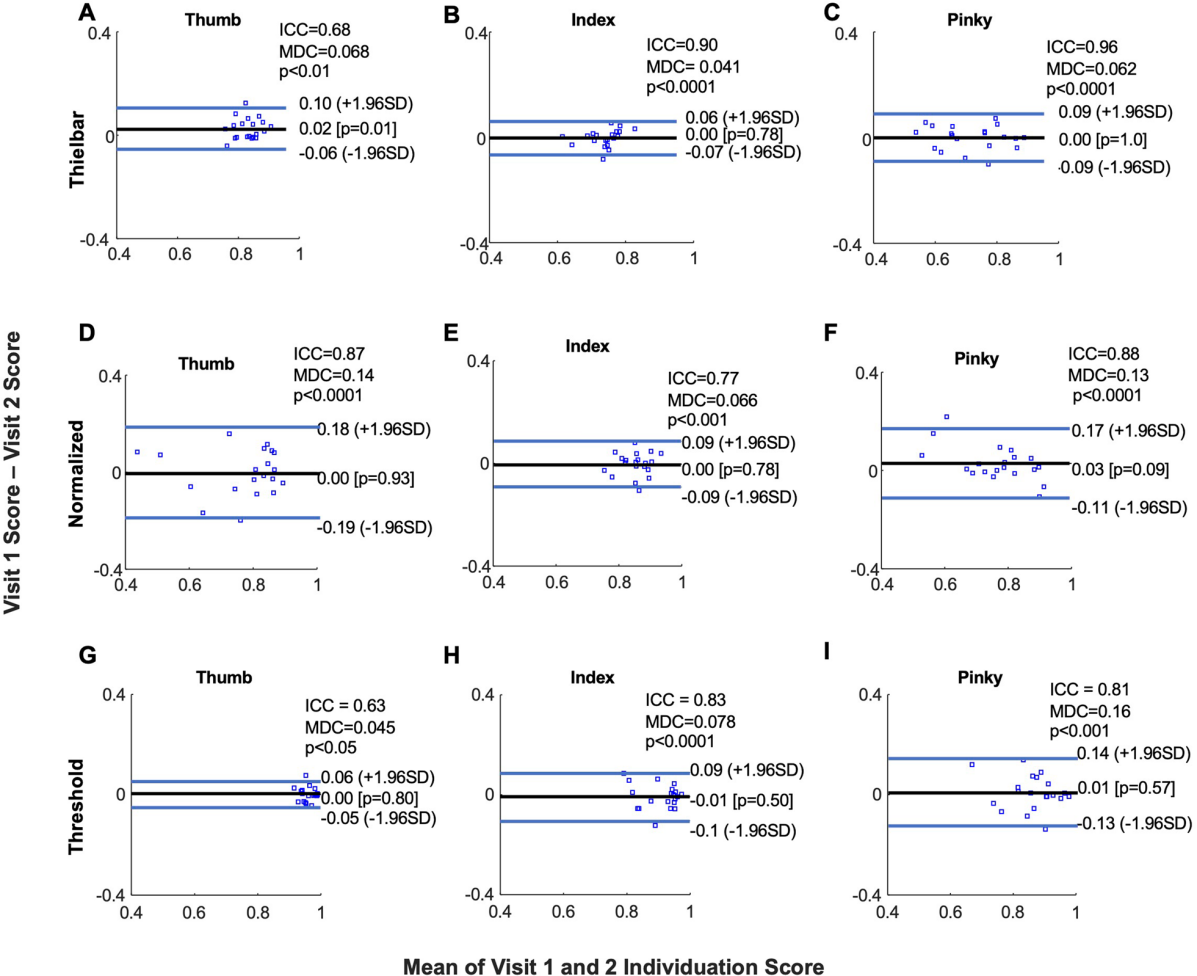
Bland Altman plots of individuation scores. The repeatability of Thielbar (**A**–**C**), Normalized (**D**–**F**), and Threshold (**G**–**I**) individuation scores can be visualized as the black line represents the mean difference of individuation scores across the two visits, and the majority of participants fall within the two blue lines representing ± 1.96 standard deviations of the mean difference. Participant-level data is represented as blue squares. Intra-class correlation coefficients (ICC), corresponding p-values, and minimal detectable changes (MDC) are shown. The thumb, index, and pinky fingers are shown here as the thumb and index were typically participants’ highest scores while the pinky was the lowest

Across the two study visits, individuation scores calculated with the Thielbar, normalized, and threshold equations were repeatable, but the strength of repeatability varied by both equation and finger (Figs. 6, 7). Minimal change in individuation scores was observed within participants for all three equations (Fig. 6).

For Thielbar individuation scores, intra-class correlation coefficients (ICC’s) were 0.68 (p < 0.01), 0.90 (p < 0.001), 0.82 (p < 0.001), 0.85 (p < 0.0001), and 0.96 (p < 0.0001) for the thumb, index, middle, ring, and pinky fingers, respectively (Fig. 7A–C). Aside from one outlier, the difference in participants’ Thielbar individuation scores fell within 1.96 standard deviations of the sample’s mean difference. The difference in participants’ Thielbar individuation scores were randomly distributed around the sample’s mean difference for the index (p = 0.78), middle (p = 0.74), ring (p = 0.58), and pinky (p = 1.0) fingers (Fig. 7A–C). However, for the thumb Thielbar individuation scores, a positive shift can be observed (p = 0.01) (Fig. 7A). The MDC for Thielbar individuation scores were 0.068, 0.041, 0.057, 0.073, and 0.062 for the thumb, index, middle, ring, and pinky fingers respectively (Fig. 7A–C). Figures 6 and 7 present data from participants’ thumb and index fingers as these were typically their highest scores as well as their pinky fingers as these were their lowest.

For normalized individuation scores, ICC’s were 0.87 (p < 0.0001), 0.77 (p < 0.001), 0.72 p < 0.005), 0.60 (p < 0.05), and 0.88 (p < 0.0001) for the thumb, index, middle, ring, and pinky fingers, respectively (Fig. 7D–F). Similar to the Thielbar individuation scores, the difference in participants’ normalized individuation scores fell within 1.96 standard deviations of the sample’s mean difference except for one outlier for each finger (Fig. 7D–F). The differences in participants’ normalized individuation scores were randomly distributed around the samples’ mean difference for the thumb (p = 0.93), index (p = 0.78), middle (p = 0.10), ring (p = 0.07), and pinky (p = 0.09) fingers (Fig. 7D–F). MDC’s were calculated as 0.14, 0.066, 0.085, 0.12, 0.13 for the thumb, index, middle, ring, and pinky fingers respectively (Fig. 7D–F).

For threshold individuation scores, ICC’s were 0.63 (p < 0.05), 0.83 (p < 0.001), 0.63 (p < 0.05), 0.76 (p < 0.01), and 0.81 (p < 0.001) for the thumb, index, middle, ring, and pinky fingers, respectively (Fig. 7G–I). The difference in participants’ mean threshold individuation scores were randomly distributed around the group’s mean difference for thumb (p = 0.93), index (p = 0.78), middle (p = 0.10), ring (p = 0.07), and pinky (p = 0.09) fingers and fell within 1.96 standard deviations of the sample’s mean difference in individuation score except for one outlier for each finger (Fig. 7G–I). MDC’s were 0.15, 0.066, 0.085, 0.12, and 0.13 (Fig. 7G–I).

### Validating individuation scores

**Fig. 8:**
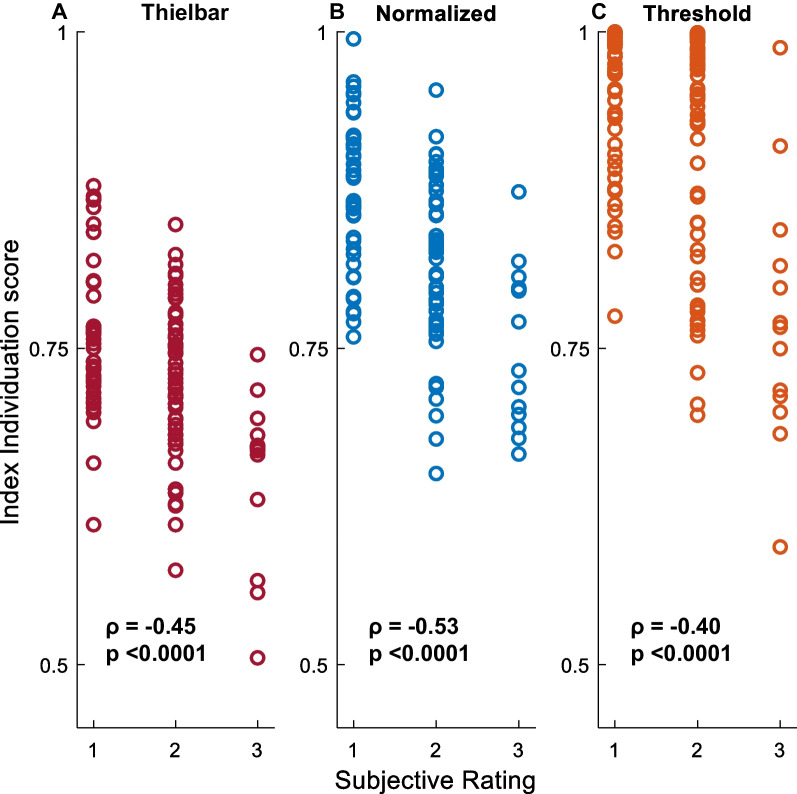
Validating individuation scores through subjective review. Index Thielbar (maroon) (**A**), normalized (blue) (**B**), and threshold (orange) (**C**) individuation scores plotted against subjective ratings of 1 (‘excellent’), 2 (‘moderate’), or 3 (‘poor’). Points represent participants’ individual trials of right-hand index individuation trials at their first study visit. Spearman’s Rho (ρ) and corresponding p-values are shown for each relationship

Although the ranges of scores within each subjective rating overlapped, it was possible to identify notable statistical relationships. For example, for index finger trials, ρ was equal to − 0.45 (p < 0.0001), − 0.53 (p < 0.0001), and − 0.40 (p < 0.0001) for the Thielbar, normalized, and threshold individuation equations, respectively (Fig. 8). For the pinky finger trials ρ was equal to − 0.66 (p < 0.0001), − 0.31 (p < 0.0001), and − 0.23 (p < 0.05) for the Thielbar, normalized, and threshold individuation equations, respectively (Fig. 8).

**Fig. 9:**
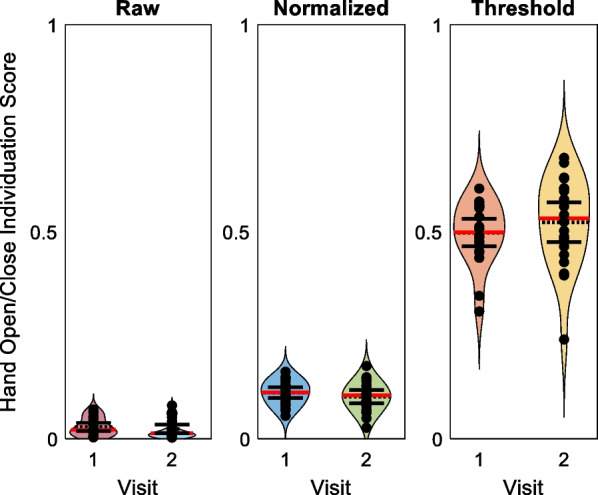
Individuation scores for hand open/close data. Thielbar (**A**), normalized (**B**), and threshold (**C**) individuation scores were calculated for trials of closing the hand into a fist and opening again at both study visits. First visits are shown on the left side of each graph in maroon, blue, and orange with second visits on the right in cyan, green, and yellow. Black circles represent participants’ mean individuation scores. Dashed horizontal black lines represent sample means, and red horizontal lines represent sample medians

For trials of participants closing their hand into a fist and opening it again, scores fell into ranges of 0.0027 to 0.080, 0.027 to 0.18, and 0.24 to 0.68 for the Thielbar, normalized, and threshold equations, respectively (Fig. 9). Overlapping 95% confidence intervals for sample’s mean individuation score between study visits for hand close/open trials is noted. Scores near zero were expected as all fingers were moving resulting in a complete lack of individuation. The threshold scores are notably higher for these trials relative to the Thielbar and normalized scores, likely because this equation calculates the displacement of the other fingers when the indicated finger reaches its 50% threshold instead of maximal displacement. Therefore, while scores of less than 0.25 are possible, they are rarely achieved with this equation (Fig. 9).

## Discussion

The purpose of this study is to establish an objective, repeatable, and quantitative platform to evaluate finger individuation that may be translatable across multiple clinical and research settings. To this end, here we provide a set of normative values for three separate finger individuation scores in healthy adults using the Cyberglove III, each of which emphasizes a different aspect of performance and, therefore, might better fit certain clinical applications.

While the data glove used here has been and continues to be used in many research settings [1, 8, 25, 27–30, 36, 37], our work enables more informed translational applications when objective measurements of finger individuation are needed. Finger individuation is a core component of basic hand dexterity that allows for the completion of other complex and dexterous tasks in everyday life. Rather than measuring the ability complete individual dexterous tasks, it can be useful to directly measure finger individuation, as it is inherent to nearly all abilities of the hand and can thus more broadly inform the extent of motor deficit.

Because each individuation score emphasizes different aspects of individuation, each is likely more applicable to certain clinical and research scenarios, as explored in the subsections below. While there is no ‘best’ individuation score, there are inherent advantages and drawbacks to each based on the equations used to calculate them.

### Thielbar individuation score

The cohort of healthy volunteers enrolled in our study predictably scored slightly higher than the cohort of patients with irreversible brain injury due to stroke in Theilbar and colleagues’ study [25] as shown in Fig. 4 of their results. While the Thielbar scores calculated in our study demonstrated overall repeatability, it varied by finger. For example, while the pinky scores were rather widely distributed, excellent repeatability was observed as the ICC was greater than 0.9 [38, 39]. There was ‘good’ repeatability of Thielbar individuation scores of the index, middle, and ring fingers as these ICC’s fit into the range of 0.75–0.9. There was ‘moderate’ repeatability of Thielbar individuation scores of the thumb as the ICC fell into the range of 0.5–0.75 [38]. There was a non-random distribution of score differences for Thielbar Thumb scores, but this was the only instance where such a finding was observed. A possible explanation is greater familiarity with the task resulting in better performance at the second visit. The MDC was low, indicating a relatively high sensitivity to change. Future studies will aim to determine the minimal clinically important difference (MCID) in patients with hand motor dysfunction, which will be critical to adapting these techniques to rehabilitative settings aimed at determining patients’ recovery trajectory.

The statistically significant relationship between Thielbar individuation scores and subjective ratings indicate that trials subjectively assessed as ‘good’ (1), ‘moderate’ (2), or ‘poor’ (3) followed a trend in Thielbar individuation score. Moreover, when the Thielbar individuation equation was used to calculate individuation scores with trials of participants closing their hand into a fist, scores were expectedly close to zero as all fingers were engaged in these trials. Ultimately, the Thielbar individuation score has been shown to be sensitive enough to detect change in patients with stroke [25], and here we show that healthy adults predictably achieve higher scores than those with pathology. However, the score does not emphasize range of motion, likely resulting in a diminished sensitivity to changes in individuals with minor dysfunction more exaggerated at end-range positions.

### Normalized individuation score

The normalized individuation equation was designed to promote full range of motion, as individuation is most difficult at the end-range [40, 41]. These scores demonstrated good repeatability of the thumb, index, and pinky fingers and moderate repeatability of the middle and ring fingers. As healthy participants were more able to reach their maximum range of motion across all trials, they achieved higher mean individuation scores. The rather low MDC’s indicate a high sensitivity to change.

Notably, there was a negative relationship between normalized individuation scores and subjective ratings as higher individuation scores were associated with the superior subjective ranking of ‘one’ while lower scores were associated with an inferior ranking of ‘two’ or ‘three’. The ranges of individuation scores within each subjective rating category clearly overlapped. Appropriately, the normalized individuation equation resulted in uniformly low scores for trials of participants closing their hand into a fist. Additionally, normalized scores spanned a wider distribution than Thielbar scores, further reflecting the effect of including range of motion in the calculation. Ultimately, the normalized score appears to be more affected by smaller performance deviations than the Thielbar equation, meaning it is likely more sensitive to detect mild dysfunction at end-ranges but less suitable for grosser motor pathology that requires a tighter set of healthy normative values for comparison.

### Threshold individuation score

For the threshold individuation equation, participants’ mean scores were the highest as this equation assesses whether participants can perform the basic components of the instructed movement. As these participants were all healthy, typical movements easily surpassed the 50% threshold. There was good repeatability of the index, ring, and pinky individuation scores, as well as moderate repeatability of thumb and middle fingers. The threshold score MDC’s were also low, similar to the Thielbar and Normalized equations. Given these results, we hypothesize that the threshold equation will prove most useful in assessing patients with severe deficits.

Similar to the Thielbar and normalized scores, a weak negative relationship was identified between individuation score and subjective rating. Additionally, the ranges of scores within each subjective rating clearly overlapped. Relationships between subjective ratings and individuation scores from all equations indicate the objective quantitative methods of assessing hand dexterity appropriately correspond to a subjective perception of performance quality. This is an important consideration as subjective methods have traditionally been used to assess dexterity in the clinical setting for many disturbances in the hand motor system [2, 9, 14].

Threshold individuation scores from hand open/close trials were higher and more widespread than the Thielbar and normalized scores for the same trials. The inability to reach a score of 0 may be an artifact of how the scores were calculated rather than a true limitation, as the order in which the fingers were closed when forming a fist was most critical in this calculation. During these trials, the first finger to reach its threshold was arbitrarily selected as the “indicated finger” so an individuation score could be calculated. Thus, if one finger reached its threshold while others remained close to their neutral position, a slightly higher threshold individuation score would result. Overall, results from the threshold equation suggest it would likely be most appropriate in assessing severe impairments, and that it would be unlikely to sensitively detect the most subtle perturbations in performance.

### Applications to clinical and research settings

As explored in the introduction, fine motor control of the hands is incredibly physiologically complex. As such, clinically, the hands are shared by multiple medical subspecialties, including neuro-, plastic, and orthopedic surgery. Authors on this manuscript represent a diverse group interested in how brain lesions and surgeries affect fine motor control [42, 43], developing neuro rehabilitation techniques to improve outcomes in patients with CNS injury [7, 44], better understanding of fine motor dysfunction from cervical myelopathy [45], and predicting and tracking outcomes of nerve transfers and extremity injuries [46]. With these applications in mind, the need to develop better techniques for quantifying hand function, especially in patients with central nervous system injury, becomes clear [47–52]. The platform tested here begins to address this gap, as results from this cohort of healthy volunteers provides a range of normative values that appear sufficiently repeatable over time for most clinical applications. Next steps will be applying this platform in pre-, intra-, and post-operative settings [53] to assess its performance in these settings with varying pathology, as well as developing related but different tests of other hand dexterity modalities (i.e., isometric finger force generation).

### Limitations

The major limitation of this work is our limited sample size and narrow demographics, as our cohort of participants consisted of20 right-handed mostly young adults, most of whom were female. Fortunately, it has been previously demonstrated that hand strength and function typically remain consistent until approximately 60 years of age [54], so future renditions of this study with middle-aged adults would likely produce similar results. Notably, extrapolations to the pediatric, elderly, and left-handed population should be made with caution or not at all. Additionally, we did not study patients with known pathology such as osteoarthritis of the hand, peripheral nerve injuries, or nerve compressions, which can confound performance [55, 56]. Therefore, extrapolation of these results to participants with musculoskeletal impairments should not be made. Although the techniques presented here were developed with the intention of assessing motor system dysfunction, the translation into assessing patients with dysfunction of any sort remains to be seen in future studies.

Second, although the results presented here depict statistical evidence for the repeatability of these methods, visual representations of scores show clear within-participant changes in individuation scores for the normalized and threshold individuation equations. It is important to note, however, that participants with large changes in individuation scores for a given finger often had similar changes for other fingers. This suggests the changes across the two visits for a participant have multiple contributory factors, such as changes in effort, familiarity with the task, and slight changes in positioning both between participants and visits. These points often appeared as outliers in the Bland–Altman plots, but all outliers were included in the analyses. This variability should be noted in case of the need for repeat testing. 

Third, while range of motion is an important component of kinematic individuation, other important components include muscle strength, speed, accuracy, and smoothness, to name a few. While these components of individuation were not directly assessed here, we plan to investigate these equally important aspects of finger movement in future studies. Additionally, some hand motor tasks do not require full range of motion, such as typing, further emphasizing the unique utility of our three individuation equations which place different weigh on range of motion as a factor in calculating and individuation score.

## Conclusions

In sum, here we provide a set of normative values for three separate finger individuation scores in healthy adults using the Cyberglove III. Each individuation score emphasizes a different aspect of finger individuation performance that may be more uniquely applicable to certain clinical scenarios. It is our hope that this platform can be used within and across centers wishing to share objective data in the physiological study of hand dexterity.

## Data Availability

All necessary data supporting these results are available in the manuscript figures and text. If readers have any further questions regarding the data and results, they can contact the corresponding author.

## Abbreviations

DIP: Distal interphalangeal
E_n_: Euclidean norm
E_ni_: Euclidean norm of the indicated finger
E_nib_: Euclidean norm of the indicated finger’s baseline position prior to the initiation of movement
E_nit_: Euclidean norm threshold of the indicated finger: 50% of the sample’s mean maximum Euclidean norm achieved by the indicated finger
E_no_: Euclidean norm of a non-indicated finger
E_nob_: Euclidean norm of a non-indicated finger’s baseline position
E_not_: Euclidean norm threshold a non-indicated finger: position of the non-indicated digit when the indicated digit reaches E_nit_
E_thresh_: Euclidean norm threshold for each of the non-indicated fingers
ICC: Intra-class correlation coefficient
M1: Motor cortex
MCID: Minimal clinically important difference
MCP: Metacarpophalangeal
MDC: Minimal detectable change
PIP: Proximal interphalangeal
